# Action Myoclonus Secondary to Donepezil: Case Report and Literature Review

**DOI:** 10.5041/RMMJ.10510

**Published:** 2023-10-29

**Authors:** Jamir Pitton Rissardo, Ana Letícia Fornari Caprara

**Affiliations:** Medicine Department, Federal University of Santa Maria, Santa Maria, Brazil

**Keywords:** Aricept, donepezil hydrochloride, drug-induced, movement disorder, myoclonus

## Abstract

Movement disorders associated with donepezil have been only rarely reported. Herein, we describe an older woman who developed myoclonus secondary to donepezil. A 61-year-old female presented with brief involuntary twitching. The patient reported that she consulted a general practitioner about 1 month before due to memory complaints. A diagnosis of mild cognitive impairment was made. Donepezil was started. After 4 weeks, she presented to our emergency department due to significant twitching. Multifocal myoclonus was observed. These movements occurred during rest and voluntary movement. Laboratory exams and cerebrospinal fluid analysis were normal. A cranial computed tomography and brain magnetic resonance imaging were unremarkable. Electroencephalography did not show epileptic activity. Electromyography revealed burst durations varying between 50 and 100 ms. Diazepam intravenous was started, which improved her abnormal movement within 1 hour. On the next day, she developed the same clinical symptoms of presentation. Donepezil was discontinued, and clonazepam was started. The patient had a complete recovery from her myoclonus. To the authors’ knowledge, there are six reports of myoclonus secondary to donepezil/galantamine. There is no report of rivastigmine-induced myoclonus. The most frequent presentation was multifocal myoclonus. The management was the discontinuation of the acetylcholinesterase inhibitor. All the individuals recovered within 3 weeks.

## INTRODUCTION

Donepezil hydrochloride, a reversible acetylcholinesterase inhibitor, was first approved for medical use by the United States Food and Drug Administration (FDA) in 1996. Its efficacy has been demonstrated in patients with dementia in all Alzheimer’s disease stages. Off-label uses of this medication include dementia with Lewy bodies, mild cognitive impairment, Parkinson’s disease dementia, and vascular dementia. The most common side effects reported with donepezil are gastrointestinal symptoms. Movement disorders secondary to donepezil hydrochloride were rarely reported in the literature.^1^ To the authors’ knowledge, there are six cases of myoclonus secondary to donepezil or galantamine.^2^–^7^ The present study is the first to describe donepezil-induced myoclonus in a patient with mild cognitive impairment. Herein, we report a case of an older woman presenting with memory complaints who was treated with donepezil. After 1 month, she developed jerking movements associated with this acetylcholinesterase inhibitor.

## CASE REPORT

A 61-year-old female presented with brief generalized involuntary twitching within 3 days of its onset. These abnormal movements occurred at rest and were provoked by voluntary movement of her extremities. She described difficulty walking due to sudden movements in her lower limbs when she began to walk. She had controlled hypertension with enalapril 10 mg, two tablets daily. Her family history was negative for neurological and psychiatric disorders.

The patient reported that about 1 month before developing this jerking movement, she had consulted with a general practitioner due to memory complaints. The primary care physician performed a mini-mental state examination (MMSE) and a Montreal cognitive assessment (MOCA), for which she scored 26 and 24, respectively. She had 8 years of formal education and worked as a lawyer. The main neurocognitive domains affected in the patient were complex attention (sustained attention), short-term memory (cued recall), and language (object naming). Laboratory tests were within normal limits, including vitamin B12, thyroid-stimulating hormone, creatinine kinase, and serologies for sexually transmitted diseases. The patient scored 4 (normal) on the geriatric depression scale (GDS). Brain magnetic resonance imaging was negative for structural abnormalities. A diagnosis of mild cognitive impairment was made. Donepezil 5 mg, one tablet daily for 4 weeks, was started.

On day 28 of donepezil therapy, the patient developed occasional jerking movements, mainly affecting her inferior limbs symmetrically. After 3 days, she presented to our emergency department due to a significant increase in the frequency and distribution of the twitching. Neurological examination showed multifocal myoclonus involving her head, face, upper extremities, and lower extremities. These movements occurred during rest and voluntary movement provoked by auditory and facial sensitive stimuli. Her muscle strength was graded 5 (normal) on the Medical Research Council (MRC) scale. Cranial nerves and sensory examination were normal. Deep tendon reflexes scored 3 on the MRC scale, which were brisk and symmetric on all sites. The remaining physical examination was unremarkable.

Laboratory exams were within normal limits. Cranial computed tomography and brain magnetic resonance imaging were normal. Cerebrospinal fluid analysis showed an opening pressure of 17 cmH_2_O, 68 mg/dL of glucose (91 mg/dL plasma glucose), no white blood cells, no red blood cells, and 40 mg/dL of protein. Electroencephalography was normal without background epileptic activity. Surface electromyoneurography assessing orbicularis oculi, biceps, and tibialis anterior revealed burst durations varying between 50 and 100 ms. Conduction velocity was approximately 50 m/s in the patient’s upper limbs.

Diazepam 10 mg intravenous was started, which improved symptoms within 1 hour. On the next day, the patient developed the same clinical manifestation of presentation. Her medications were revised. Based on previous literature reports, donepezil was discontinued, and clonazepam 0.5 mg, one tablet daily for 7 days, was started. The patient had a complete recovery from her abnormal movements. No new medication was attempted to manage her memory complaint. After further questioning, she reported that this was not affecting her daily living activities. She did not report any abnormal movement in the follow-up at 1 and 3 months. An investigation for autoimmune, primary neoplasia, and paraneoplastic disorder was negative.

## DISCUSSION

Donepezil, galantamine, and rivastigmine are drugs classified as reversible acetylcholinesterase inhibitors. The mechanism of action of these medications involves inhibition of the acetylcholinesterase enzyme leading to increased levels of the neurotransmitter acetylcholine, which in turn enhances cholinergic neurotransmission.^1^ Also, they can upregulate nicotinic receptors in the cortical neurons, promoting a neuroprotective environment. Therefore, it is believed that donepezil and other acetylcholinesterase inhibitors improve cognition due to increased acetylcholine concentrations in the hippocampus, raising hippocampal theta rhythm amplitude elicited by stimulation of the brainstem reticular formation.^8^

There are only a few case reports of myoclonus secondary to acetylcholinesterase inhibitors. We identified six cases after a review of the English-language literature, and we compared them with the present case (Table 1).^2^–^7^ A literature search was performed in Embase, Google Scholar, Lilacs, Medline, Scielo, and ScienceDirect, using a set of terms that included myoclonus, donepezil, rivastigmine, and galantamine.

**Table 1:** Case Reports of Myoclonus Secondary to Acetylcholinesterase Inhibitors.

Reference	Drug	Age/Sex	Comorbidities	Clinical Manifestations	Management	Follow-up
Colebatch and Bacsi (2007)^2^	Galantamine	69/F	Dementia, mitral valve prolapse, atrial fibrillation, depression, macular degeneration, benign colonic polyps, transient ischemic attack	Multifocal myoclonus affecting upper and lower limbs	NA	NA
Hernández-Fernández et al. (2011)^3^	Galantamine	80/M	Alzheimer’s disease, hypertension, dyslipidemia, and chronic renal failure	Respiratory myoclonus, also known as diaphragmatic flutter	Galantamine was discontinued; valproic acid and clonazepam were started	After 3 weeks, he was asymptomatic
Luna (2012)^4^	Galantamine, 4 months	87/M	Dementia	Multifocal myoclonus	Galantamine was discontinued	Movement symptoms resolved 2 weeks later
Bougea et al. (2014)^5^	Donepezil, 25 days	80/F	Alzheimer’s disease	Myoclonus in both upper and lower extremities	Donepezil was discontinued	Myoclonus resolved within 36 hours
Whateley et al. (2018)^6^	Donepezil, 7 weeks	93/F	Alzheimer’s disease, hypertension	Multifocal myoclonic non-synchronous, affecting all four limbs at rest	Donepezil was discontinued	Myoclonus resolved within 2 days
Amlang et al. (2019)^7^	Donepezil, 4 weeks	67/M	Occipital strokes, hypertension, diabetes mellitus type 1, hepatitis C	Multifocal myoclonic movements of head, face, upper extremities, and trunk	Donepezil was discontinued; clonazepam was started	Myoclonic movements subsided within 1 day
Present case (2023)	Donepezil, 4 weeks	61/F	Mild cognitive impairment, hypertension	Multifocal myoclonus of distal limbs and face; myoclonus worsened with action	Donepezil was discontinued; clonazepam was started	Myoclonus subsided within 1 day

F, female; M, male; NA, not available/not reported.

In 1987, Mayeux et al. reported the first case of myoclonus secondary to an acetylcholinesterase inhibitor.^9^ They described two patients with Alzheimer’s disease for whom physostigmine was prescribed. The first patient had increased myoclonus frequency with increasing physostigmine doses. Also, the authors described a dose adjustment in the second individual leading to a complete recovery of motor symptoms. These two reports are interesting because they provide different explanations for physostigmine-associated myoclonus. One reveals a significant dose-dependent response, and the other a threshold effect of an all-or-none mechanism. As a result, physostigmine is no longer prescribed due to neurotoxicity and high parasympathomimetic activity.

Ten years after the above reports with physostigmine, Abilleira et al. described a patient with middle-stage Alzheimer’s disease who presented with action myoclonus following tacrine use.^10^ They observed a recurrence of the twitching with tacrine rechallenge. In 2013, tacrine was withdrawn by the FDA due to safety concerns.

Currently, the most common acetylcholinesterase inhibitors prescribed for Alzheimer’s disease are donepezil, rivastigmine, and galantamine. Table 1 describes the case reports published related to myoclonus secondary to acetylcholinesterase inhibitors. There is no report of myoclonus secondary to rivastigmine. The mean age of the individuals that developed myoclonus was 76 years. Females were more frequently affected (57%). The majority of individuals had at least one comorbidity. The most frequent indication for acetylcholinesterase inhibitors was Alzheimer’s disease. Other indications were unspecified dementia and mild cognitive impairment. Myoclonus was multifocal in almost all cases, at presentation. Hernández-Fernández et al. described an interesting report of respiratory myoclonus, also known as diaphragmatic flutter, following galantamine therapy.^3^ Management consisted of discontinuation of the drug in all subjects. In some cases, benzodiazepines were prescribed, showing a shorter period of improvement of the motor symptoms compared to those who did not receive benzodiazepines. All the individuals recovered within 3 weeks.

**Figure 1:**
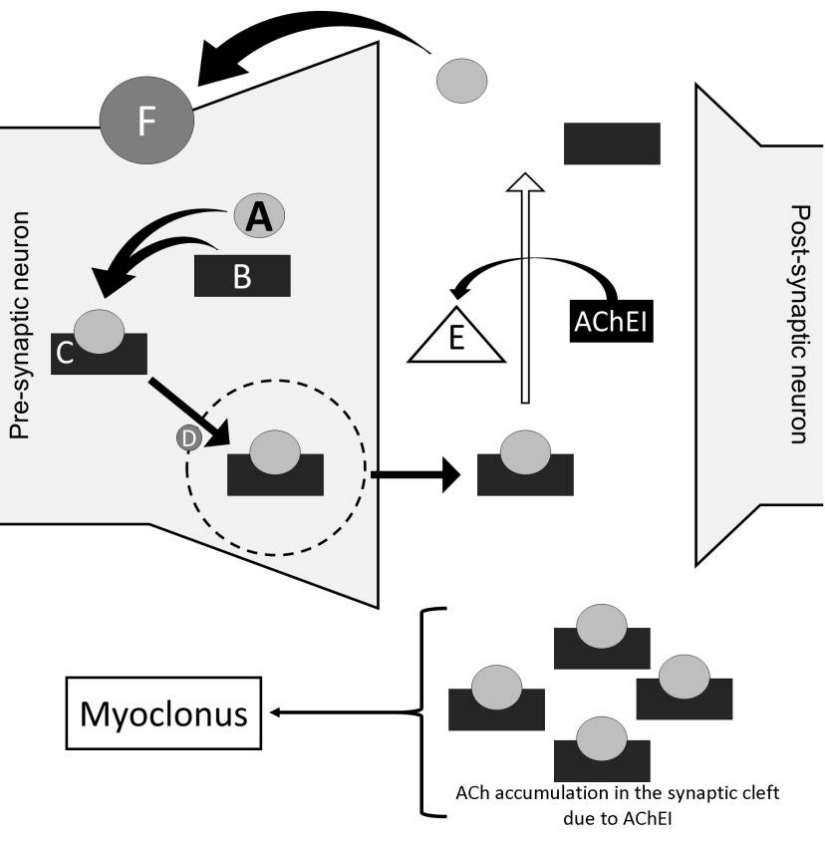
Possible Pathophysiological Hypothesis for Explaining Myoclonus Secondary to Donepezil. The inhibition of AChE by the AChEI leads to the accumulation of ACh in the synaptic cleft. In predisposed brain regions, this increased cholinergic concentration can lead to twitching. A, choline; B, acetate; C, acetylcholine; D, acetylcholine carrier; E, acetylcholinesterase (AChE); F, choline carrier; Ach, acetylcholine; AChEI, acetylcholine-sterase inhibitor (donepezil, galantamine, and rivastigmine).

Myoclonus secondary to acetylcholinesterase inhibitors is probably related to a primary cholinergic mechanism (Figure 1). In experimental studies, the reduction of acetylcholine function leads to improvement of picrotoxin-induced myoclonus.^6^ So, high levels of acetylcholine may be associated with involuntary twitching frequency. Also, donepezil increased extracellular dopamine release in the cortex and dorsal hippocampus in rat models. Surprisingly, norepinephrine levels in the prefrontal cortex were unchanged.^1^ Most cases occurred with donepezil and less frequently with other acetylcholinesterase inhibitors. This can be explained by the fact that galantamine and rivastigmine, compared to donepezil, have higher affinity and selectivity for acetylcholinesterase, preventing cholinergic adverse events at therapeutic doses.^6^,^8^

## CONCLUSION

Myoclonus secondary to donepezil was rarely reported in the literature. Myoclonus most frequently develops within the first month of acetylcholinesterase therapy, and complete recovery is seen with the discontinuation of the drug. Clinicians should be aware of this uncommon association because patients affected by cognitive impairment have a high percentage of comorbidities leading to possible misdiagnoses.

## Abbreviations

FDA: United States Food and Drug Administration
GDS: geriatric depression scale
MMSE: mini-mental state examination
MOCA: Montreal cognitive assessment
MRC: Medical Research Council
